# “Barriers beneath the surface”: healthcare access and cultural beliefs affecting cervical cancer screening among egyptian women: a qualitative study

**DOI:** 10.1186/s12905-026-04911-3

**Published:** 2026-10-17

**Authors:** Marwa Mamdouh Shaban, Nermen Abdelfatah Mohamed, Mostafa Shaban

**Affiliations:** 1 Community Health Nursing Department, Faculty of Nursing, Cairo University, Cairo, Egypt; 2 Adult Nursing Department, Faculty of Nursing, Kafrelsheikh University, Kafrelsheikh, Egypt; 3 Gerontological Nursing Department, Faculty of Nursing, Cairo University, Cairo, Egypt

**Keywords:** Cervical cancer screening, Cultural beliefs, Qualitative study, Egyptian women, Healthcare access, Barriers, Community health nursing, Reflexive thematic analysis

## Abstract

**Background:**

Cervical cancer remains the fourth most common cancer among women globally, with disproportionate burden concentrated in low- and middle-income countries. In Egypt, participation in cervical cancer screening remains critically low, shaped by intersecting layers of socio-cultural belief, limited health literacy, and structural barriers in healthcare access. Despite growing quantitative evidence, the lived experiences of Egyptian women navigating these barriers remain poorly understood. This study explored the healthcare access factors and cultural beliefs affecting cervical cancer screening among Egyptian women attending a gynaecology outpatient clinic.

**Methods:**

A qualitative study using reflexive thematic analysis (RTA) was conducted at the gynaecology outpatient clinic of Kafrelsheikh University Hospital, Kafrelsheikh, Egypt, between October 2025 and February 2026. Purposive sampling was used to recruit 22 adult Egyptian women. Data were collected through in-depth semi-structured interviews conducted in Arabic, audio-recorded, and transcribed verbatim. Analysis followed Braun and Clarke’s six-phase reflexive thematic analysis framework.

**Results:**

Four overarching themes were identified: (1) “Silenced by Stigma and Shame” — the role of taboo, modesty norms, and social censure in suppressing women’s willingness to seek screening; (2) “The Body as a Site of Cultural Negotiation” — how religious beliefs, fatalistic attitudes, and patriarchal permission structures governed bodily access and health-seeking; (3) “When the System Stands in the Way” — structural healthcare barriers including cost, unavailability of female providers, and limited facility infrastructure; and (4) “Navigating Between Fear and Faith” — the psychological tension between fear of cancer diagnosis and religious reliance on divine will.

**Conclusions:**

Cervical cancer screening avoidance among Egyptian women is rooted in a complex convergence of cultural norms, patriarchal structures, religious fatalism, and systemic healthcare inadequacies. Culturally sensitive interventions, nurse-led outreach, and expanded access to female healthcare providers are urgently needed. Community health nurses are positioned as a critical bridge between women’s health needs and culturally acceptable care.

**Supplementary Information:**

The online version contains supplementary material available at https://doi.org/10.1186/s12905-026-04911-3.

## Introduction

### Reviewer 1 comment

“The paper is interesting and adds a new perspective to the literature on screening for gynecological cancers. However, the authors did not make a strong case for the necessity of routine screening for cervical cancer in a country with very low prevalence of this disease as per GLOBOCAN 2024 statistics for Egypt. In the meantime, the authors make strong statements criticizing the health care system for not making cervical cancer screening services accessible to women, when the Ministry of Health has other more pressing priorities for women’s health (e.g. breast cancer screening, reducing maternal mortality among other). On a related note, the authors do not provide any contextual information on cervical cancer screening services in Egypt (e.g. location, cost etc.).”

#### Response

Cervical cancer remains a formidable global public health challenge, ranked the fourth most common cancer in women worldwide. According to GLOBOCAN 2024, an estimated 662,044 new cervical cancer cases and 348,709 deaths occurred in 2022, with an age-standardised incidence rate of 14.12 per 100,000 women globally [1].While GLOBOCAN 2024 indicates a low age-standardized incidence rate for cervical cancer in Egypt relative to global figures, the absence of a centralized cancer registry and systematic screening suggests potential underreporting. Thus, opportunistic screening and risk stratification remain essential. Currently, cervical cancer screening services in Egypt are primarily opportunistic, localized within tertiary/academic centers or private clinics, and largely reliant on out-of-pocket payments, limiting rural and low-income accessibility. Mortality burdens are overwhelmingly concentrated in low- and middle-income countries (LMICs), where approximately 94% of deaths occur, reflecting profound inequities in access to prevention, screening, and treatment [2]. This epidemiological disparity is particularly pronounced in the Eastern Mediterranean Region (EMR), where cervical cancer is the sixth most common malignancy among women and where participation in structured screening programmes remains low across most member states [3]. Egypt, despite its status as the most populous nation in the Arab world, lacks a universal national cervical cancer screening programme, contributing to late-stage presentations and poor survival outcomes [4].

### Reviewer 1 comment

“The authors did not provide statistics on the prevalence of cervical cancer in Egypt.”

#### Response

Recent GLOBOCAN 2024 estimates indicate that cervical cancer remains an important health burden among Egyptian women, with approximately 1,302 new cases and 820 deaths estimated in 2022. The age-standardised incidence and mortality rates were 2.8 and 1.8 per 100,000 women, respectively [5]. These figures underscore the continued importance of strengthening cervical cancer prevention and screening efforts in Egypt, particularly in relation to women’s awareness, accessibility of services, and culturally influenced healthcare-seeking behaviours.

### Reviewer 1 comment

“Finally, the authors should have started the paper with findings on women’s knowledge of cervical cancer and their awareness of screening tests for cervical cancer. On several occasions, the authors argue that there is stigma around cervical cancer screening in Egypt when the link between cervical cancer and HPV is unlikely to be known to the public.”

#### Response

Existing research suggests that knowledge and awareness of cervical cancer and its prevention among Egyptian women remain variable. A nationwide cross-sectional study involving 897 Egyptian women reported substantial gaps in awareness of cervical cancer screening, with lack of knowledge about the screening procedure (79.8%), unfamiliarity with the screening site (64.2%), and financial burden (53.2%) identified among the most reported barriers [4]. Other studies have similarly identified limited knowledge of cervical cancer risk factors, symptoms, prevention, and screening among Egyptian women [6, 7]. Awareness of human papillomavirus (HPV), the primary causal factor for cervical cancer, may also be limited, suggesting that women may make screening decisions without a clear understanding of the relationship between HPV infection and cervical cancer. However, limited knowledge does not necessarily explain all reluctance towards screening. Concerns about gynecological examination, embarrassment, privacy, cultural expectations, and perceptions of healthcare services may influence women’s decisions independently of their understanding of HPV. Qualitative exploration is therefore important for understanding how women interpret cervical cancer, screening, and related healthcare experiences within their social and cultural context.

### Reviewer 2 comment

“The paper could be improved by properly integrating a theoretical perspective in the introduction section.”

#### Response

To conceptualize the complex determinants of cervical cancer screening engagement in this population, this study was guided by the Health Belief Model (HBM). The HBM posits that individuals’ health-related actions are shaped by their perceptions of susceptibility to a condition, perceived severity of the disease, perceived benefits of taking preventive action, and perceived structural or psychological barriers. In addition, cues to action and self-efficacy play pivotal roles in prompting health behaviors. Applying the HBM as an interpretive theoretical lens allowed us to systematically explore how socio-cultural norms, knowledge deficits, fear of diagnosis, and healthcare access constraints intersect to influence Egyptian women’s screening behaviors and decisions.

Nurses—and community health nurses in particular—occupy a uniquely strategic position in addressing the cervical cancer screening gap in LMICs. As primary-contact health professionals working within communities, they are positioned to deliver health education, facilitate access to services, dispel misconceptions, and provide culturally tailored counselling to women who may otherwise remain outside the healthcare system [6]. In Egypt, community health nurses serve as frontline advocates in outpatient and primary healthcare settings, and their capacity to engage women on sensitive reproductive health topics is central to any population-level screening effort. Yet their effectiveness is constrained by the same barriers that prevent women from accessing care: persistent stigma around gynecological examination, religious and cultural taboos, low awareness of cervical cancer etiology, and inadequate screening infrastructure [4, 7].

Existing quantitative studies have documented low knowledge levels, negative attitudes, and high barrier scores around cervical cancer screening among Egyptian women. A nationwide cross-sectional study involving 897 Egyptian women found that lack of knowledge about the procedure (79.8%), unfamiliarity with the screening site (64.2%), and financial burden (53.2%) were the most cited barriers [4]. Similarly, studies from other Muslim-majority societies have demonstrated that cultural modesty norms, male gatekeeping of women’s health decisions, and religious fatalism significantly mediate screening uptake [8, 9]. However, quantitative surveys are inherently limited in capturing the lived experiential reality of these barriers—the embodied, relational, and ideological dimensions through which women come to understand and navigate the decision to seek or avoid screening. This qualitative gap is pronounced within the Egyptian context, where limited research has explored the subjective meanings, women attach to cervical cancer screening and the social processes through which avoidance is enacted and sustained.

Beyond individual-level factors, structural dimensions of healthcare access are equally implicated. The availability and affordability of screening services, the gender composition of available providers, waiting times, and institutional responsiveness to women’s cultural preferences all shape engagement with preventive care. In conservative Muslim societies such as Egypt, the availability of female gynecologists is not merely a matter of patient preference but of fundamental acceptability of care—a prerequisite without which many women will not attend regardless of awareness or motivation [8, 10]. The interplay between these structural realities and deeply embedded cultural scripts creates a multi-layered barrier landscape that qualitative methodology is particularly suited to illuminate.

This study therefore aimed to explore, in depth, the healthcare access factors and cultural beliefs that affect cervical cancer screening engagement among Egyptian women attending a gynecology outpatient clinic in Kafr Elsheikh Governorate. The research question was: How do Egyptian women perceive and experience healthcare access and cultural factors in relation to cervical cancer screening, and how do these perceptions shape their screening behaviors and decisions?

### Reviewer comment

"I pointed out to the authors several locations where criticism of the health care system was unsubstantiated".

#### Response

We have reviewed each of the statement’s revisions are as follows:

1. Statement regarding inadequate screening infrastructure:
  We have added supporting evidence from published literature and revised the wording to avoid implying a universal deficiency across the Egyptian healthcare system.
2. Statement regarding affordability and availability of screening services:
  We have added appropriate supporting references and clarified that these factors have been reported as barriers in specific populations and settings rather than presenting them as universally applicable characteristics of the Egyptian healthcare system.
3. Statement regarding institutional responsiveness to women’s cultural preferences:
  We have moderated the wording and now present this as a potential healthcare-access factor identified in literature rather than as an established deficiency of the healthcare system.

Overall, we have ensured that statements describing healthcare-system barriers are either supported by appropriate evidence or framed as participants’ reported experiences and perceptions where they arise from the qualitative findings.

## Methods

### Research design

A qualitative study design using reflexive thematic analysis (RTA) was selected to explore the complex subjective and social dimensions of cervical cancer screening avoidance. Qualitative inquiry is epistemologically appropriate for research questions concerned with meaning, perception, and lived experience, and RTA — as developed by Braun and Clarke [11] — provided a robust, flexible framework for the systematic identification, analysis, and interpretation of patterns across rich interview data. Unlike coding reliability approaches, reflexive TA foregrounds the researcher’s active analytic role and generates theoretically grounded, interpretive themes rather than simply descriptive categories [11, 12]. The constructivist paradigm underpinning this study posits that women’s understandings of cervical cancer and screening are co-constructed through cultural, relational, and institutional contexts rather than residing as pre-existing facts to be discovered.

#### Reviewer 2 comment

“The authors should include a qualitative reporting guideline that they followed in developing their report.”

#### Response

This study was conducted and reported in strict accordance with the Consolidated Criteria for Reporting Qualitative Research (COREQ) 32-item checklist (see Additional File 2).

### Study setting and recruitment

The study was conducted at the gynaecology outpatient clinic of Kafrelsheikh University Hospital, one of the major tertiary referral hospitals in Kafrelsheikh Governorate, Nile Delta region, Egypt. The clinic serves a diverse catchment area encompassing both urban and rural populations, making it an appropriate site for exploring heterogeneous perspectives on screening barriers. Recruitment was conducted through purposive sampling, targeting women who represented variation in age, educational attainment, marital status, residence (urban/rural), and prior screening experience. The lead researcher (N.A.M.) approached eligible women in the clinic waiting area, provided verbal and written information about the study in Arabic, and obtained written informed consent prior to any data collection.

### Sample size and adequacy

A total of 22 women participated in the study. Rather than applying the potentially misleading concept of data saturation — which implies a fixed and discoverable endpoint inconsistent with the interpretive epistemology of reflexive TA [12] — sample adequacy was assessed using the information power framework [13]. According to Malterud et al. [13], a sample with narrow aim, specific participant characteristics, established theory, high-quality dialogue, and cross-case analysis requires fewer participants to yield sufficient information power. The present sample satisfied these criteria: the research aim was clearly bounded, participants were drawn from a defined clinical setting, theoretical grounding in health behaviour and cultural competence literature was strong, and data were rich and nuanced. Recruitment continued until the research team judged that the data corpus provided sufficient depth and breadth to address the research question.

### Inclusion and exclusion criteria

Women were eligible to participate if they (1) were adult Egyptian females aged 21 years or older, (2) were attending the gynaecology outpatient clinic at the time of recruitment, (3) were able to communicate in Arabic, and (4) provided written informed consent. Women were excluded if they had a current diagnosis of cervical cancer or cervical dysplasia (which would confound experiences related to screening), if they were critically ill, or if they declined participation.

#### Reviewer 1 comment

“Methods for recruiting research participants should be described in more detail.”

#### Response

The women who attending the gynaecology outpatient clinic during the recruitment period were screened against the predefined inclusion and exclusion criteria. Eligible women were approached by a member of the research team, who explained the study purpose, procedures, voluntary nature of participation, and confidentiality arrangements. Women were given an opportunity to ask questions and consider participation before providing written informed consent. Participation was entirely voluntary, and declining participation had no effect on the care they received. Recruitment continued until the research team considered the resulting dataset to have sufficient information power to address the research question.

### Data collection

Individual in-depth semi-structured interviews were conducted by the primary researcher (N.A.M.) between October 2025 and February 2026 at a private consultation room within the hospital to ensure confidentiality. All interviews were conducted in Arabic, the participants’ mother tongue, to facilitate authentic and unguarded expression. Interview duration ranged from 35 to 65 min (mean 48 min). All interviews were audio-recorded with explicit participant consent. The interviewer maintained a reflective field journal throughout data collection to document emerging analytic observations, contextual notes, and positionality reflections.

### Interview structure and conduct

The interview guide was developed iteratively based on the research question, a review of relevant literature, and consultation between all three authors. It comprised seven thematic domains: general health practices; awareness and knowledge of cervical cancer; knowledge of screening procedures; cultural beliefs and social influences; healthcare access and barriers; personal attitudes and fears; and recommendations. Each domain contained four to six open questions accompanied by probes to encourage elaboration (see Additional File 1 for the full interview guide). The guide was piloted with three participants prior to commencement; minor wording adjustments were made, and pilot interviews were included in the final corpus as no substantive revisions were necessary.

### Recording and transcription

All audio recordings were transcribed verbatim in Arabic by the primary researcher and subsequently translated into English by a bilingual nursing researcher not otherwise involved in the study. Back-translation of a 20% random sample was conducted to verify semantic accuracy and cultural equivalence. Transcripts were shared with participants for member checking; two participants requested minor clarifications, which were incorporated.

### Data analysis

Data analysis followed the six phases of Braun and Clarke’s reflexive thematic analysis [10]: (1) familiarisation with the data through repeated reading of transcripts and reflective notetaking; (2) generation of initial codes applied systematically across the dataset; (3) construction of initial theme candidates through sorting and collating codes; (4) reviewing and refining themes to ensure internal coherence and distinction between themes; (6) defining and naming themes with analytic precision; and (7) producing the final report. Analysis was conducted using NVivo 14 to manage coding and facilitate systematic comparison across cases. All three authors participated in the analytic process, with regular discussion meetings used to interrogate emerging interpretations and resolve differences in coding judgement.

### Ethical considerations

Ethical approval was granted by the Scientific Research Ethics Committee of Kafrelsheikh University, Egypt (Reference Number: KFSIRB200-1003; approval date: 30 March 2026). The study was conducted in full accordance with the principles of the Declaration of Helsinki [14]. Written informed consent was obtained from all participants before interview commencement, covering the purpose of the study, the voluntary nature of participation, the right to withdraw at any time without consequence, and data confidentiality. No identifiable data were shared beyond the research team. All audio recordings and transcripts were stored on password-protected encrypted devices accessible only to the authors.

### Rigour and reflexivity

Trustworthiness was established through multiple strategies aligned with Lincoln and Guba’s criteria. Credibility was enhanced through prolonged engagement at the research site, member checking with participants, and researcher triangulation across three authors. Transferability was supported through thick description of the study context, participant characteristics, and analytic process. Dependability was ensured through detailed documentation of methodological decisions in an audit trail. Reflexivity was operationalised through the lead researcher’s field journal, in which the team documented positionality statements. Specifically, the first author (N.A.M.) is an Egyptian woman with professional nursing experience in women’s health, whose insider position facilitated participant rapport but required conscious bracketing of assumptions about cultural norms and expected responses.

#### Reviewer 2 comment

“The authors should describe the techniques they used to enhance trustworthiness and rigor.”

#### Response

To establish methodological rigor and qualitative quality, Lincoln and Guba’s criteria for trustworthiness (credibility, transferability, dependability, and confirmability) were systematically embedded across the study design:

- Credibility: Credibility was enhanced through prolonged engagement at the research site, enabling the research team to understand the institutional and social context of the clinic. Researcher triangulation was operationalized through independent transcript coding by two authors (N.A.M. and M.M.S.), followed by consensus-building discussions with the third author (M.S.). Member checking was performed with participants to verify transcript accuracy and semantic translation.
- Transferability: Transferability was supported by providing a detailed, thick description of the study setting, sociocultural environment, inclusion criteria, and participant demographic and clinical characteristics, enabling readers to evaluate the applicability of findings to similar low-resource or conservative cultural contexts.
- Dependability: Dependability was maintained through a meticulous audit trail documenting all operational and analytical decisions, including raw transcript excerpts, codebook development, initial theme groupings, and meeting notes reflecting team discussions.
- Confirmability: Confirmability was operationalized through reflexivity practices. The lead interviewer maintained a reflective field journal during data collection to track personal reactions, emerging assumptions, and potential positionality biases. The first author (N.A.M.), as an Egyptian female nurse with clinical experience in women’s health, utilized an insider position to establish rapid rapport with participants, while consciously bracketing pre-existing clinical assumptions during thematic coding.

## Results

### Characteristics of participants

Twenty-two women participated in the study. Table 1 presents the sociodemographic characteristics of participants.

**Table 1:** Sociodemographic characteristics of participants (*N* = 22)

Characteristic	Category	*n*	%
Age (years)	21–30	6	27.3
	31–40	8	36.4
	41–50	5	22.7
	> 50	3	13.6
Marital Status	Married	18	81.8
	Single	2	9.1
	Widowed/Divorced	2	9.1
Educational Level	Illiterate/Primary	4	18.2
	Secondary/Vocational	9	40.9
	University/Postgraduate	9	40.9
Residence	Urban	11	50.0
	Rural	11	50.0
Employment	Employed	10	45.5
	Housewife	12	54.5
Previous Pap Smear	Yes	5	22.7
	No	17	77.3
Health Insurance	Insured	14	63.6
	Uninsured	8	36.4

### Findings

Analysis of interview data generated four overarching themes, each comprising two sub-themes. Table 2 provides a summary of these themes and sub-themes.

**Table 2:** Themes and sub-themes identified from reflexive thematic analysis

Theme	Sub-themes	Core Description
Theme 1: “Silenced by Stigma and Shame”	1.1 Reproductive shame and gendered silence 1.2 Fear of community judgment and social exposure	Cultural taboo surrounding gynaecological examination leads women to suppress health-seeking behaviour to avoid social stigma.
Theme 2: “The Body as a Site of Cultural Negotiation”	2.1 Religious fatalism and divine dependence 2.2 Patriarchal gatekeeping of health decisions	Women’s bodies are governed by religious belief systems and male authority, positioning cervical screening as culturally transgressive.
Theme 3: “When the System Stands in the Way”	3.1 Structural and financial barriers to access 3.2 Absence of gender-concordant and empathetic care	Healthcare system inadequacies, including cost, provider gender, and quality of service, create a hostile environment for screening uptake.
Theme 4: “Navigating Between Fear and Faith”	4.1 Fear of diagnosis and anticipatory dread 4.2 Religious coping as both shield and barrier	Psychological ambivalence between cancer-related anxiety and religious acceptance produces complex screening avoidance and approach behaviours.

#### Theme 1: “Silenced by stigma and shame”

The first theme captures the pervasive cultural silence surrounding women’s reproductive and gynaecological health in the Egyptian context. Women across the sample described a deeply internalised norm of shame — an expectation that matters pertaining to the reproductive body were private, unspoken, and potentially dishonourable to discuss in public or clinical spaces. This silence was not merely private but was actively enforced through social surveillance, peer censure, and familial disapproval, creating a culture in which approaching cervical cancer screening was itself constructed as a morally suspect act. The theme encompassed two sub-themes: reproductive shame and gendered silence, and fear of community judgment and social exposure.

##### Reproductive shame and gendered silence

Many participants described a deep-seated reluctance to discuss anything connected to their reproductive anatomy, rooted in modesty norms instilled from childhood. For some women, the mere act of disclosing that they had undergone a gynaecological examination was perceived as immodest. The word “shame” — in Arabic, haya — appeared repeatedly and was understood not merely as embarrassment but as a moral virtue tied to honour, femininity, and religious propriety.

*“Since I was a child*,* we were taught never to speak about these parts of the body. Even going to a gynaecologist feels like something you should hide from people. How would I explain to my mother-in-law that I went for a Pap smear? She would think something was wrong with me morally.” (P04)*.

This comment illustrates how social relationships — particularly intergenerational female relationships — actively police women’s engagement with gynaecological care. The mother-in-law represents a key figure in the surveillance network through which cultural norms are transmitted and enforced.

*“When my husband found out I was referred for a smear*,* he was very upset. He said*,* “Why does a doctor need to examine you there?” He did not understand it was for my health. To him*,* it was about honour.” (P11)*.

This account foregrounds how shame and honour are not simply internalised by women themselves but are projected onto them by male partners, demonstrating the embodied, relational nature of the barrier.

*“I know I should go but every time I think about it*,* I feel too embarrassed. It is a very private area. Even telling a female doctor feels wrong somehow. We are raised to cover*,* not to show.” (P17)*.

##### Fear of community judgment and social exposure

Beyond personal modesty, women expressed considerable anxiety about the social consequences of being known to have undergone cervical cancer screening. In community contexts where gossip circulates through extended kinship networks, a visit to a gynaecology clinic was interpreted not as preventive healthcare but as evidence of sexual disease or moral impropriety.

*“People in our village would talk. If someone saw me coming out of the women’s clinic*,* they would start saying things — that maybe I have a disease*,* or that there is something bad in my marriage. I cannot afford for people to think that about me.” (P02)*.

The fear of reputational damage was particularly pronounced among rural participants and those from tightly knit neighbourhood communities. This social exposure risk created a rational calculation in which the perceived costs of screening — community stigma, marital instability, social ostracism — outweighed any anticipated health benefit.

*“My neighbour went for a smear test and people started spreading rumours about her. From that day*,* I decided I will never go. My reputation is more important than any test.” (P08)*.

“Even at the clinic, I was worried someone would recognise me in the waiting area. I kept thinking — what if a relative sees me? What would they tell people?” (P20).

#### Theme 2: “The body as a site of cultural negotiation”

The second theme illuminates how women’s bodies — specifically their reproductive bodies — became contested terrain in which religious beliefs, cultural norms, and patriarchal authority intersected to determine whether cervical screening was permissible, necessary, or even meaningful. Women did not approach their bodies as autonomous sites of individual agency but as relational entities governed by divine will and male authority. Two sub-themes were generated: religious fatalism and divine dependence, and patriarchal gatekeeping of health decisions.

##### Religious fatalism and divine dependence

A prominent explanatory framework among participants was the Islamic concept of tawakkul — trusting in God’s will — which for some women justified non-participation in preventive healthcare on the grounds that illness and health are divinely determined. This did not reflect an absence of faith in medicine generally, but rather a specifically gendered application of religious acceptance to intimate bodily health practices, particularly those involving gynaecological examination.

*“Allah decides when someone gets sick and when they recover. I pray*,* I eat well*,* and I trust in God. Why would I go for a test to look for a disease I do not have? If Allah wills it*,* no test will protect me anyway.” (P06)*.

This perspective reflects a form of health fatalism that, while not uniquely Islamic, is particularly strongly culturally embedded in conservative Egyptian contexts. It positions preventive screening as potentially inconsistent with spiritual surrender, making religious reframing a necessary component of any behaviour change intervention.

*“Our prophet*,* peace be upon him*,* said the body is an amanah — a trust. But some of the older women in my family say that seeking tests is like not trusting in God. I am confused because I also know nurses say we should go.” (P14)*.

“My aunt told me that everything is written. If I am meant to get cancer, a smear will not stop it. God knows best. This is what I believed for a long time.” (P19).

##### Patriarchal gatekeeping of health decisions

For married women, husbands played an authoritative and frequently restrictive role in health-seeking decisions. Many women described needing explicit spousal permission to attend gynaecological appointments, and several reported that this permission had been withheld or conditionally granted. Male partners’ objections were grounded in a combination of jealousy, honour-protection norms, and medical mistrust.

*“My husband says he does not want any male doctor to examine me*,* ever. Even for a smear. He says he would rather I stay at home than be examined by a man. And at our clinic*,* there is no female gynaecologist available every day.” (P03)*.

The intersection of spousal permission and the structural absence of female providers created a compounded barrier that was particularly difficult for women to navigate individually. Even women who personally desired screening reported being unable to act on this desire without their husband’s approval.

*“I wanted to go last year. But my husband said no. He was not angry — he just did not think it was necessary. He said*,* “You are not sick*,* why are you looking for problems?” I could not go against his decision.” (P09)*.

"In our culture, the husband decides health matters. I cannot just go alone. I need him toagree, to accompany me sometimes, to pay. If he does not support it, it does not happen."(P16)

#### Theme 3: “When the system stands in the way”

The third theme addresses structural and systemic barriers within the Egyptian healthcare environment that impede cervical cancer screening access. These barriers were not merely logistical inconveniences but constituted fundamental exclusions for women with limited resources, restrictive social circumstances, or specific provider requirements. Two sub-themes were identified: structural and financial barriers to access, and absence of gender-concordant and empathetic care.

##### Structural and financial barriers to access

Economic constraints emerged as a consistent and powerful deterrent to screening participation. For women from lower socioeconomic backgrounds — many of whom were uninsured or underinsured — the cost of a Pap smear, including transportation, registration fees, and laboratory charges, was prohibitive. This finding is consistent with national data showing that financial burden is among the most commonly cited barriers to cervical screening in Egypt [4].

*“The smear itself is not expensive*,* but by the time you add the bus fare to the city*,* the registration fee*,* the laboratory fee*,* and time off work*,* it becomes a lot. My husband would say we cannot afford it this month. And then next month the same. It never happens.” (P07)* .

For rural women in particular, geographic distance and transportation challenges compounded financial barriers, creating a situation in which attendance required significant investment of time, money, and social capital.

*“I live forty minutes from here by microbus. I would need to come in the morning*,* wait for hours*,* and come back. Who takes care of my children? Who pays for the bus? There is no screening centre in our village. Everything is far.” (P01)*.

“I tried to go last Ramadan but the clinic only opens on certain days. When I arrived, the queue was so long I left. I thought: this is not worth the trouble. They are not making it easy for us.” (P13).

##### Absence of gender-concordant and empathetic care

The availability and attitude of healthcare providers was a critical determinant of women’s willingness to engage with screening services. The majority of participants stated categorically that they would only accept a gynaecological examination from a female provider. In settings where female gynaecologists were unavailable or not consistently present, many women declined attendance entirely. Beyond gender concordance, women described experiences — both personal and reported through social networks — of dismissive, hurried, or judgmental treatment from healthcare providers that actively discouraged return visits.

*“I went once*,* three years ago. The doctor — a man — did not explain anything. He just told me to lie down. I felt humiliated. I cried on the bus home. I have not gone back since.” (P05)*.

This account illustrates how a single negative clinical encounter, particularly involving violation of gender norms, can produce lasting disengagement from preventive services. The emotional aftermath of this experience was palpable and clearly shaped subsequent help-seeking behaviour.

*“My friend told me she was shamed by a nurse who said*,* “Why didn’t you come earlier?” — as if she was to blame. After that*,* I decided I would not go. They make you feel guilty instead of supported.” (P10)*.

“I need a female doctor who speaks to me kindly, who explains everything. Just someone who treats me like a human being. That is not too much to ask. But I have not found that.” (P21).

#### Theme 4: “Navigating between fear and faith”

The fourth theme reflects the psychological ambivalence experienced by women as they negotiated between cancer-related fear and religiously mediated acceptance. This theme captures an internal conflict that was not simply a matter of knowledge deficit but of deeply affective, spiritually anchored reasoning about illness, mortality, and the limits of human intervention. Two sub-themes emerged: fear of diagnosis and anticipatory dread, and religious coping as both shield and barrier.

##### Fear of diagnosis and anticipatory dread

A substantial number of participants identified fear — not of the procedure itself but of what the procedure might reveal — as a primary deterrent to seeking screening. This diagnostic dread operated as a form of protective avoidance: by not testing, women preserved a state of uncertain but functional health and avoided confronting the possibility of cancer. This psychologically self-protective logic, while understandable, carried profound public health consequences.

*“What if they find something? Then what? I would have to tell my family. Treatment costs money we don’t have. My children would worry. Sometimes I think it is better not to know. If I feel okay*,* I am okay.” (P15)*.

This participant’s account crystallises the rationality of avoidance within a context of economic precarity and limited social support for serious illness. The prospect of diagnosis was inseparable from its social and financial consequences, making non-participation a coherent, if health-compromising, strategy.

*“I am terrified of cancer. Just the word makes me tremble. So I stay away from anything that might bring that word into my life. I know this is not logical but I cannot help it.” (P18)*.

“When my sister was diagnosed with a different cancer, I watched what the family went through. I decided I never want that for my family. So I do not go for tests. If it is there, knowing will only bring pain earlier.” (P22).

##### Religious coping as both shield and barrier

While religious faith was identified in Theme 2 primarily as a fatalistic constraint, this sub-theme captures its more dynamic role as a coping mechanism that could both inhibit screening (by positioning illness acceptance as virtuous) and — in a smaller number of cases — motivate it (through the Islamic obligation of self-care as an expression of gratitude for the body entrusted by God). This nuanced finding underscores the heterogeneity of Islamic health belief and the importance of avoiding monolithic characterisations of religion as simply a barrier.

*“I was afraid*,* yes. But I also read in the Quran that we should take care of the body Allah gave us. Eventually I thought: screening is not doubting God*,* it is being a good steward. My imam agreed. That is why I came today.” (P12)*.

This participant’s account represents a reframing of religious duty that opened a pathway to screening. The role of religious leaders — imams — as potential allies in health promotion is suggested by this account and represents an important avenue for intervention.

*“My prayers give me peace when I am worried. But I also know that Allah gave us medicine for a reason. I try to balance both. I pray and I go to the doctor. They are not opposites.” (P03)*.

“Sometimes I think: if I get sick, Allah will help me. Other times I think: take the test, be responsible. I go back and forth. I have not decided yet.” (P20).

### Thematic summary: relationships across themes

The four themes are not independent but form an interlocking system of barriers operating at individual, relational, institutional, and cultural levels. Theme 1 (stigma and shame) establishes the cultural architecture of silence within which all health-seeking decisions are made. Theme 2 (cultural negotiation) demonstrates how this silence is actively maintained through religious frameworks and patriarchal authority. Theme 3 (systemic barriers) shows that even where cultural motivation exists, structural healthcare failures prevent engagement. Theme 4 (fear and faith) reflects the affective and spiritual negotiations women must individually conduct in the absence of supportive systems. Together, these themes reveal that cervical cancer screening avoidance in this context is neither ignorance nor indifference but a socially rational, culturally embedded, psychologically protective response to a genuinely hostile environment for women’s preventive healthcare.

## Discussion

This qualitative study provides the first in-depth exploration of healthcare access and cultural belief barriers to cervical cancer screening among Egyptian women using a reflexive thematic analysis framework. The four themes generated — stigma and shame, cultural negotiation of the body, systemic healthcare barriers, and the interplay of fear and faith — offer a nuanced and contextually rich account that extends and deepens the existing literature.

### Reviewer 1 comment

“Finally, the authors should have started the paper with findings on women’s knowledge of cervical cancer and their awareness of screening tests for cervical cancer. On several occasions, the authors argue that there is stigma around cervical cancer screening in Egypt when the link between cervical cancer and HPV is unlikely to be known to the public.”

#### Response

The findings demonstrate that baseline awareness of cervical cancer and its screening options remain limited among participants. This aligns with broader regional literature indicating that public understanding of Human Papillomavirus (HPV) as an underlying cause of cervical cancer is low. Consequently, socio-cultural barriers to screening in this setting are generally not driven by STI-associated stigma. Rather, screening hesitancy stems primarily from cultural norms regarding modesty, anxiety or discomfort associated with invasive pelvic examinations, and the widespread perception that gynecological consultations are only necessary when severe symptoms are present.

The pervasive shame and stigma identified in Theme 1 resonates strongly with findings from other Muslim-majority societies. Khan and Woolhead [8], in a qualitative study among Muslim women in Dubai, identified modesty and honour norms as central mediators of screening avoidance, with women describing gynaecological examination as culturally transgressive and potentially dishonourable. Similarly, Namoos et al. [9] found that conservative Muslim American women of Egyptian and Arab descent identified cultural and social norms as the primary barriers to cervical cancer prevention behaviours, with shame and community surveillance functioning as powerful disincentives. The Egyptian-specific contribution of the present study lies in its documentation of how intergenerational surveillance — particularly the mother-in-law as enforcer of reproductive silence — operates as a unique social mechanism for maintaining the barrier, one not prominently described in existing literature from non-Egyptian Arab contexts.

Theme 2 extends existing understanding of religious fatalism in cancer screening behaviour. While fatalism has been documented as a barrier in diverse Islamic contexts [10, 15], the present study identified its heterogeneous expression: for most women, tawakkul functioned as a legitimising framework for non-participation; for a minority, Islamic self-care ethics provided a reframing of screening as spiritual duty. This finding aligns with Christie-de Jong et al. [15], who demonstrated in a faith-based intervention for Muslim women in Scotland that religiously tailored messages could shift health beliefs by positioning screening as consistent with rather than contrary to Islamic values. The implication for Egypt is significant: community health nurses working with Islamic scholars and local imams may be able to reframe screening uptake as a religious obligation, potentially mobilising religious motivation for health behaviour change.

The structural barriers identified in Theme 3 echo a pattern well-documented across the Eastern Mediterranean Region. A systematic review of cervical cancer screening barriers across EMR countries [3] categorised individual, health-system, and social structural factors and found that financial burden, distance, provider unavailability, and negative provider attitudes consistently deterred participation. The nationwide Egyptian study by Behery et al. [4] found that cost and procedural unfamiliarity were the most prevalent barriers in a sample of 897 women. The present study adds qualitative depth to these statistics by documenting the affective consequences of negative clinical encounters — particularly how a single experience of gender norm violation or provider dismissiveness could produce lasting, generalised disengagement from preventive services. This underscores the critical role of nursing communication skills, empathy, and gender-concordant care provision in sustaining — not merely initiating — screening participation [16].

Theme 4, capturing the tension between diagnostic fear and religious coping, reflects patterns identified in the broader qualitative literature on cancer avoidance. The concept of diagnostic dread — avoiding testing to preserve a state of protective ignorance — has been described in diverse cultural contexts and is not unique to Muslim-majority societies. However, its intersection with religious acceptance (tawakkul) in this study produced a particularly complex psychological landscape, where women were simultaneously afraid of knowing and spiritually equipped to accept uncertainty. Community health nurses are positioned to address both poles of this tension: providing clear, compassionate information about the treatability of early cervical abnormalities (reducing catastrophic anticipation) while engaging respectfully with religious frameworks to support rather than undermine women’s spiritual coping resources.

### Limitations

This study has several limitations that warrant acknowledgement. The study was conducted at a single tertiary hospital site in Kafrelsheikh Governorate, which may limit transferability to other Egyptian regions with different sociodemographic profiles or healthcare infrastructure. The purposive sample, while diverse in key characteristics, is not representative of the full heterogeneity of Egyptian women. As is inherent in qualitative inquiry, the findings reflect the interpretive lens of the research team, whose prior knowledge and professional backgrounds inevitably shape analysis; reflexivity strategies were employed to mitigate but cannot entirely eliminate this influence. Interviews were conducted within a clinical setting, which may have introduced social desirability effects despite assurances of confidentiality. Additionally, data were collected between October 2025 and February 2026, and rapidly evolving national health initiatives may have shifted the context since that time.

### Implications for nursing practice, education, and policy

#### Reviewer 2 comment

“The authors should consider including the concept of cultural humility as part of their implications for nurses.”

#### Response

The findings carry immediate and actionable implications across nursing practice, education, and health policy. At the clinical level, nurses must move beyond basic cultural competence toward practicing cultural humility. Cultural humility requires nurses to maintain a posture of ongoing self-reflection, acknowledge inherent power imbalances between providers and patients, and respect patients’ unique cultural backgrounds without judgment. In the context of gynecological care in Egypt, practicing cultural humility means actively recognizing women’s modesty concerns, validating their psychological dread, and engaging in transparent, empathetic dialogue rather than imposing authoritative clinical agendas. Pre-screening education sessions led by female nurses who embody cultural humility within community health settings could effectively destigmatize screening before women arrive at the clinical encounter.

Nurse-led community outreach programmes, particularly in rural areas, could address the geographic and financial barriers that prevent access, while mobile screening units and home-visit initiatives may reach women unable to attend institutional settings. At the educational level, nursing curricula should embed content on cultural safety, Islamic health beliefs, and cervical cancer screening communication, equipping graduates to navigate these consultations competently. At the policy level, the findings support a strong case for: (1) development of a national cervical cancer screening programme in Egypt with standardised free or subsidised Pap smear provision; (2) mandatory female provider availability at all gynaecology outpatient clinics; (3) engagement of religious community leaders in cervical cancer awareness campaigns; and (4) integration of cervical cancer screening into routine community nursing visits.

### Conclusion

Cervical cancer screening avoidance among Egyptian women is not a product of ignorance or apathy but a socially rational, culturally embedded response to a healthcare environment that fails to adequately accommodate the intersection of gender, culture, religion, and structural access. The four themes generated in this study — shame and stigma, cultural negotiation of the body, systemic barriers, and the tension between fear and faith — collectively map a barrier landscape that must be addressed holistically if screening participation is to increase. Community health nurses are not peripheral to this challenge but central: as the healthcare profession most intimately positioned within communities, and best equipped to navigate the cultural and relational dimensions of women’s health, nurses must be empowered with resources, training, and system-level support to lead Egypt’s cervical cancer prevention response.

## Supplementary Information

Supplementary Material 1.

## Data Availability

The datasets generated during the current study are not publicly available due to the sensitive nature of the qualitative interview data and the need to protect participant confidentiality. De-identified data excerpts are available from the corresponding author on reasonable request.
