# Supplementary material for: “Barriers beneath the surface”: healthcare access and cultural beliefs affecting cervical cancer screening among egyptian women: a qualitative study
Source: BMC Womens Health. 2026 Oct 17;26:531. doi: 10.1186/s12905-026-04911-3 (PMC13675963; doi:10.1186/s12905-026-04911-3)
Supplement: Supplementary file 1 [file 12905_2026_4911_MOESM1_ESM.docx]

**ADDITIONAL FILE 1**

**Semi-Structured Interview Guide**

**Study Title:** "Barriers Beneath the Surface": Healthcare Access and Cultural Beliefs Affecting Cervical Cancer Screening Among Egyptian Women: A Qualitative Study

**Study Design:** Qualitative study — Reflexive Thematic Analysis (Braun & Clarke, 2006; 2021)

**Setting:** Gynaecology Outpatient Clinic, Kafrelsheikh University Hospital, Egypt

**Ethics Reference:** KFSIRB200-1003, Kafrelsheikh University Scientific Research Ethics Committee

**Language:** Interviews conducted in Arabic; transcripts translated into English with back-translation verification

**PART A: General Instructions for the Interviewer**

1. Conduct all interviews in a private, quiet, and confidential space within the clinical setting.

2. Introduce yourself, explain the study purpose, emphasise voluntary participation, and obtain signed informed consent before proceeding.

3. Use open, non-leading questions. Allow pauses. Do not interpret or anticipate responses.

4. Use probes flexibly to encourage depth — the probes listed below are guides, not scripts.

5. Adopt a neutral, non-judgmental stance throughout. This is particularly important given cultural sensitivities around reproductive health topics.

6. If a participant becomes distressed, pause the interview, offer support, and give her the choice to continue, take a break, or stop.

7. Write reflective notes in the field journal after each interview regarding your observations, emerging ideas, and positionality reflections.

**PART B: Pre-Interview Consent and Opening Checklist**

Before beginning the interview, complete the following:

**[ ]** Participant has received written information sheet in Arabic

**[ ]** Participant has signed written informed consent form

**[ ]** Participant is aware that the interview will be audio-recorded

**[ ]** Participant understands they can withdraw at any time without consequence

**[ ]** Audio recorder is functioning and participant has given consent to record

**[ ]** Assign participant pseudonym code (P01–P30) before recording begins

Opening statement (read aloud in Arabic):

*"Thank you very much for agreeing to participate in this study. I am a nursing researcher, and I am interested in understanding women's experiences and views about cervical health and cancer screening. There are no right or wrong answers — I want to hear your genuine thoughts and feelings. Everything you share will remain strictly confidential. Please feel free to speak freely."*

**PART C: Interview Guide — Questions and Probes**

The interview guide is structured across seven domains. Questions should be asked in a conversational manner; the order may be adapted to the natural flow of the conversation.

| **Domain** | **Q#** | **Interview Question** | **Probes / Interviewer Notes** |
| --- | --- | --- | --- |
| **Domain A General Health Practices (Warm-up)** | A1 | Can you tell me about how you usually look after your health? | How often do you visit a doctor? What prompts you to seek care? Do you attend regular check-ups? |
|  | A2 | What factors influence whether you decide to seek medical care? | Is it symptoms? Cost? Family advice? Distance? Time? |
|  | A3 | Have you ever attended a hospital or clinic for a preventive health check — not because you were ill? | What was that experience like? What made you decide to go? |
|  | A4 | How would you describe healthcare services available to you? | Are they accessible? Affordable? Trustworthy? Do you feel comfortable there? |
| **Domain B Awareness & Knowledge** | B1 | Have you ever heard of cervical cancer? | If yes — what do you know about it? If no — what have you heard about cancers affecting women? |
|  | B2 | What do you think causes cervical cancer? | Where did you get that information? Family? Social media? Nurse/doctor? School? |
|  | B3 | Do you think cervical cancer is common among women in Egypt? | Why do you think that? Do you know anyone affected? |
|  | B4 | Where do you usually get health information? | TV? Mobile phone? Doctor? Pharmacist? Friends/family? Religious sources? |
| **Domain C Screening Knowledge** | C1 | Have you heard about cervical cancer screening tests, like a Pap smear or HPV test? | What do you know about what these tests involve? How they are done? |
|  | C2 | Who do you think should get screened, and at what age? | Is it for married women only? For all women? Is age a factor? |
|  | C3 | Have you ever had a cervical cancer screening test? | If yes — what was that experience like? If no — what has prevented you from going? |
|  | C4 | What would help women to better understand cervical cancer screening? | Awareness campaigns? Nurse-led sessions? Religious guidance? Community events? |
| **Domain D Cultural Beliefs & Social Influences** | D1 | What do women in your community generally think about cervical cancer screening? | Are people aware of it? Do they discuss it? Is there stigma? |
|  | D2 | Are there cultural or religious beliefs that might affect women's decisions about getting screened? | What beliefs have you heard? How do they shape decisions? Does religion play a role? |
|  | D3 | How do feelings of modesty (haya) or embarrassment affect women's decisions about screening? | Is the physical examination a concern? Is gender of the doctor important? |
|  | D4 | Do family members — husband, mother, in-laws — influence a woman's decision to go for screening? | Who has the most influence? In what direction — supportive or discouraging? |
|  | D5 | Is there any stigma around cervical cancer or going for screening in your community? | What do people say? What might they think about a woman who goes? |
| **Domain E Healthcare Access & Barriers** | E1 | If you wanted to get screened, where would you go? | Do you know where to go? Is it nearby? What would you do first? |
|  | E2 | How easy or difficult would it be to access cervical cancer screening services? | Distance? Cost? Waiting time? Available appointments? Female provider? |
|  | E3 | What challenges might prevent women from getting screened? | Probe: cost, transport, time off work, childcare, lack of female doctor, waiting lists, awareness |
|  | E4 | How would you describe your experience with healthcare services in general? | Have you felt treated with respect? Kindness? Do you trust providers? |
| **Domain F Personal Attitudes & Fears** | F1 | How do you personally feel about the idea of undergoing cervical cancer screening? | Afraid? Willing? Indifferent? What feelings come up? |
|  | F2 | What concerns or fears do you have about getting screened? | Fear of the result? Fear of pain? Fear of others knowing? Embarrassment? |
|  | F3 | What would encourage you to go for a cervical cancer screening test? | Free service? Female doctor? Health campaign? Family support? Nurse recommendation? |
|  | F4 | What would discourage you? | Any past negative experience? Social concerns? Religious reasons? |
| **Domain G Nursing & Healthcare Provider Role** | G1 | Has a doctor or nurse ever discussed cervical cancer screening with you? | What did they say? Was it helpful? How did it make you feel? |
|  | G2 | How do you think healthcare providers influence women's decisions about screening? | Do nurses make a difference? What kind of communication works best? |
|  | G3 | What type of communication or support from a nurse would make you feel comfortable enough to attend for screening? | Private conversation? Community session? Home visit? Mobile phone message? |
|  | G4 | What do you think nurses could do differently to help more women attend for screening? | Training? Outreach? Speaking at mosques or community centres? House visits? |

**PART D: Closing the Interview**

Use the following questions to draw the interview to a close:

1. "Is there anything else you would like to tell me that we have not yet discussed?"

2. "What do you think is the single most important thing that would help more women attend for cervical cancer screening in Egypt?"

3. "How did you find participating in this interview today? Is there anything you would want me to change for future participants?"

Thank the participant warmly. Remind her of her right to review her transcript. Provide a copy of the study information sheet and researcher contact details. End the audio recording.

**PART E: Demographic Data Collection (Post-Interview)**

Collect the following data after the interview using a structured form (not recorded):

Age (years):

Marital status: (Single / Married / Divorced / Widowed)

Educational level: (Illiterate or primary / Secondary or vocational / University or postgraduate)

Employment status: (Employed / Housewife / Student / Retired)

Place of residence: (Urban / Rural)

Number of children (if applicable):

Health insurance status: (Insured / Uninsured)

Previous cervical cancer screening: (Yes / No) If yes, how many years ago?

**PART F: Probing Techniques Reference Card**

The following probes may be used flexibly throughout the interview to elicit deeper reflection:

"Can you tell me more about that?"

"What did that feel like for you?"

"Why do you think that is?"

"Can you give me an example?"

"What happened after that?"

"How did others around you react?"

"Has that experience changed how you think about screening now?"

"What would need to be different for your feelings about this to change?"

**Ethics and Confidentiality Statement**

This interview guide is a research instrument approved by the Scientific Research Ethics Committee of Kafrelsheikh University (Reference: KFSIRB200-1003). All data collected using this guide are subject to strict confidentiality protocols. Participant pseudonym codes shall be used in all transcripts and publications. Audio recordings shall be stored on password-protected encrypted devices and deleted at the conclusion of the study per the ethics protocol.
